# Implication of haematophagous arthropod salivary proteins in host-vector interactions

**DOI:** 10.1186/1756-3305-4-187

**Published:** 2011-09-28

**Authors:** Albin Fontaine, Ibrahima Diouf, Nawal Bakkali, Dorothée Missé, Frédéric Pagès, Thierry Fusai, Christophe Rogier, Lionel Almeras

**Affiliations:** 1 Unité de Parasitologie - UMR6236 - IFR48, Antenne Marseille de l'Institut de Recherche Biomédicale des Armées (IRBA), Le Pharo, BP 60109, 13 262 Marseille Cedex 07, France; 2 Laboratoire de Génétique et Evolution des Maladies infectieuses, UMR 2724 CNRS/IRD, Montpellier, France; 3 Unité d'Entomologie Médicale, Antenne Marseille de l'Institut de Recherche Biomédicale des Armées (IRBA), Le Pharo, BP 60109, 13 262 Marseille Cedex 07, France; 4 Institut Pasteur de Madagascar, B.P. 1274, Ambohitrakely, 101 Antananarivo, Madagascar

## Abstract

The saliva of haematophagous arthropods contains an array of anti-haemostatic, anti-inflammatory and immunomodulatory molecules that contribute to the success of the blood meal. The saliva of haematophagous arthropods is also involved in the transmission and the establishment of pathogens in the host and in allergic responses. This survey provides a comprehensive overview of the pharmacological activity and immunogenic properties of the main salivary proteins characterised in various haematophagous arthropod species. The potential biological and epidemiological applications of these immunogenic salivary molecules will be discussed with an emphasis on their use as biomarkers of exposure to haematophagous arthropod bites or vaccine candidates that are liable to improve host protection against vector-borne diseases.

## Review

During the course of evolution, haematophagy has arisen many times in disparate arthropod taxa. Between the taxa, this feeding habit has evolved independently over several million years [1,2] leading to morphophysiological differences among haematophagous arthropods. At the molecular level, this is reflected by the existence of a variety of pharmacologically active molecules in arthropod saliva used to face the constraints of vertebrate host haemostasis, inflammation and adaptive immunity [3-5].

The saliva of haematophagous arthropods is also responsible for causing allergic responses in human hosts, which are manifested by cutaneous pruritic wheal-and-flare reactions at the bite site [6,7]. Thus, a high density of haematophagous arthropods can directly affect human populations worldwide due to their presence and physical nuisance [8,9]. Beside this direct effect, arthropods can also indirectly affect human health by transmitting pathogens. Indeed, many viral, bacterial, and eukaryotic pathogens have found haematophagous arthropods ideal vectors to accomplish transmission among vertebrates. Usually, a long-lasting co-speciation has led to specific associations between pathogens and vectors [2]. Hence, pathogens often depend on few related species of vectors for transmission (Table 1). Some of these pathogens have even taken advantage of the immunomodulatory properties of haematophagous salivary proteins in order to enhance their infectivity in the vertebrate host [10,11].

**Table 1:** Taxonomic classification of major vector-borne diseases

Vectors			Diseases
Order	Family	Genius	
Diptera	Culicidae	*Anopheles*	Malaria,
			Lymphatic filariasis
		*Culex*	West Nile disease
			Japanese encephalitis
		*Aedes*	Yellow fever
			Chikungunya
			Dengue
	Psychodidae	*Phlebotomus*	Leishmaniasis
		*Lutzomyia*	
	Glossinidae	*Glossina*	Human African Trypanosomiasis
	Simulidae	*Simulium*	Onchocerciasis
	Tabanidae	*Tabanus*	Loiasis

Hemiptera	Reduviidae	*Triatoma*	Chagas disease

		*Rhodnius*	
Ixodida	Ixodidae	*Amblyomma*	Rickettsiosis
			Tularemia
		*Ixodes*	Lyme disease
			Babesiosis
		*Haemaphysalis*	Tularemia
			Tick borne encephalitis
	Argasidae	*Ornithodoros*	Relapsing fever

The taxonomic classification of the major hematophagous arthropod vectors described in the present review is given with their corresponding diseases.

Arthropod-borne diseases are a major health problem worldwide. They cause serious impacts on the economy and survival of human populations living mainly in tropical and sub-tropical countries [12-14]. To a lesser extent, human populations in developed countries are also exposed to a variety of vector-borne pathogens [15-17]. Pathogen vaccine and prophylactic drug research have so far produced little to protect individuals from many arthropod-borne diseases. Currently, vaccines are only available for the yellow fever virus [18], the Japanese encephalitis virus [19], the Rift valley fever virus [20] and the tick-borne encephalitis virus [21]. Protection against *Plasmodium*, the malaria parasites, still relies on the use of prophylactic drugs and is hampered by the escalation of drug-resistance [22].

Thus, the primary mechanism to protect individuals from vector-borne diseases is the prevention of bites from infected arthropods. This can be achieved by a combination of personal protective measures and vector control strategies adapted to vector behaviour [23-26]. These methods have been historically successful in reducing [27-29] or eliminating [30,31] the transmission of some vector-borne diseases. Currently, the effectiveness of anti-vectorial measures and the evaluation of the transmission of arthropod-borne diseases are determined by laboratory bioassay tests [32-35], by measuring the incidence, morbidity or mortality of vector-borne diseases in controlled clinical trials in the field [36,37] or by entomological methods [38,39]. Concerning mosquito-borne diseases, the entomological reference method to measure vector density is by catching landing mosquitoes on humans, which provides a good estimate of the average number of bites per person per day received from one particular vector species [40]. However, in terms of execution and supervision, this method is very laborious and dependent on the skills of the collector. In addition, the deliberate exposure of human volunteers to vectors has raised some ethical issues against this technique. As the human bite rate was shown to vary within small geographic areas [41,42], the results of local catches cannot be extrapolated to larger areas. Additionally, results from the human landing catch performed by adults can be difficult to extrapolate to children. Alternative entomological methods exist to capture medically important haematophagous arthropods, such as carbon dioxide dry ice traps, light traps and odour baited traps (to collect flying dipterans) [43] or the drag-flag method (to collect ticks) [44]. However, these tools do not differentiate anthropophilic from zoophilic arthropods and cannot precisely assess the contact between haematophagous arthropods and host. Hence, the development of new indicators and methods to evaluate the effectiveness of anti-vectorial strategies at the individual level is necessary.

A common feature shared among arthropod vectors is their habit of feeding on blood involving the injection of saliva into the host's skin. One consequence of the injection of salivary proteins is the eliciting of host antibody responses against these pharmacologically active components [45-51]. Such observations suggest that these antigenic components could potentially be used as immunological tools to evaluate individual exposure to arthropod bites.

This present survey is particularly concerned with the current knowledge of antibody responses to the salivary proteins of haematophagous arthropods. Immunogenic salivary proteins of haematophagous arthropods were first studied for their allergenic properties. However, there is strong evidence for their application in improving host protection against some vector-borne diseases and for their use as alternative immunological tools to assess individual exposure to haematophagous arthropod bites. An overview of the pharmacological activity of the main salivary proteins characterised in various haematophagous arthropod species will first be presented to provide a better understanding of the role of saliva in host defence, including haemostasis and the immune response.

## Blood-feeding behaviour among haematophagous arthropods

The phylum Arthropoda represents the vast majority of metazoan life forms on earth, with a species richness estimated at 5-10 million [52]. The blood-feeding habit has arisen and evolved independently in more than 14,000 species from 400 genera and five orders in the arthropod taxonomy [1,53]. These independent adoptions of haematophagy during the evolution of arthropod vectors required morphological, behavioural and biochemical adaptations in order to remove blood from the skin of vertebrate hosts. Indeed, blood is not an easy access nutrient due to its cryptic nature in addition to the host's behavioural and biological defensive response.

Mouthparts have adapted the following two strategies to obtain blood from vertebrates: (i) lacerating dermal capillaries and collecting the nutritive fluid in a hemorrhagic pool (pool feeding or telmophagy, *e.g*., flies from the families Tabanidae and Psychodidae and Ixodoidae (ticks) and (ii) by directly inserting mouthparts into a capillary (capillary feeding or solenophagy, *e.g*., Culicidae (mosquitoes) and Reduviidae (bugs). The development of these blood-feeding habits may have occurred in several different ways. A prolonged and close association between terrestrial hosts and arthropods that regularly fed on dead parts of the host's body or organic debris associated with the nests or burrows may have gradually established more profound parasitic relations characterised by switching to a haematophagous diet. Alternatively, the development of haematophagy would have been facilitated in some capillary feeders by morphological preadaptation of their ancestors to phytophagy or entomophagy [2,54-56].

Other important blood-feeding behaviours are displayed among haematophagous arthropods, including the duration of blood-feeding (which can range from few minutes for Culicidae [57] to several days for ticks [58]), the rate of anthropophily [59] or the obligate versus facultative haematophagous diet [56]. These singularities among different haematophagous arthropods, adding to other behavioural or biological features, such as the length of the extrinsic incubation period (time between the acquisition of an infectious agent by a vector and its ability to transmit it to other vertebrate hosts) [60], the nycthemeral activity [61] or the reproductive strategies (K- and r- selected arthropods) [62], are additional features that may have significant implications on disease transmission and on the implementation of anti-vectorial strategies. However, whichever blood-feeding strategies or feeding behaviour is used, each adoption of haematophagy requires solutions to counteract vertebrate host haemostatic, inflammatory and immune responses. At the molecular level, haematophagous arthropods have also developed, by an evolutionary process, an important diversity of pharmacological compounds in their saliva in order to prevent these physiological responses.

## The role of saliva in blood-feeding

### Salivary components and host's haemostasis

Haemostasis is a host cellular and molecular response that prevents blood loss from a damaged vessel through several redundant processes, such as blood vessel vasoconstriction, formation of a primary platelet plug (primary haemostasis) or vessel strengthening by blood coagulation (secondary haemostasis) [For review: [63,64]].

Damage to blood vessel endothelium first results in vasoconstriction that decreases blood flow at the bite site to limit the haemorrhage. Two strategies are employed by haematophagous arthropods to prevent this phenomenon. Some arthropods display salivary components which block host vasoconstrictor agents, such as peroxidase from the *Anopheles albimanus *mosquitoes [65]. Other arthropods have strong vasodilators in their saliva. For example, *Lutzomyia longipalpis *sand flies and *Simulium vittatum *black flies express maxadilan and *Simulium vittatum *erythema protein (SVEP) in their saliva, respectively, which are the most potent known vasodilators [66,67]. These two species are pool feeders that require strong vasodilatory substances to increase blood flow perfusion in superficial regions of the skin. Closely related species can use separate mechanisms to counteract vascular compression as illustrated by *Phlebotomus *sand flies that express adenosine and 5'AMP vasodilators instead of maxadilan in their saliva [68].

Vascular injuries due to the penetration of arthropod mouthparts in the host skin are also accompanied by the activation of platelets, which aggregate within seconds to form a haemostatic plug using fibrinogen as a connecting agent [63,69]. Convergent paths of evolution have lead to similar molecules in different arthropod species that inhibit or scavenge a panel of platelet-aggregating factors (Additional file 1 Figure 1). Among them, apyrase, an enzyme that hydrolyses ADP released by damaged cells and activated platelets, is ubiquitously found in the saliva of various haematophagous arthropods [70-79]. Distinct salivary proteins from a single species could present redundant effects, as illustrated by the *Rhodnius prolixus *bug saliva, which contains both a salivary apyrase and a protein named *Rhodnius prolixus *aggregation inhibitor 1 (RPAI-1) that inhibits platelet aggregation by direct binding to ADP [71,80]. More diversified molecules targeting other platelet aggregation agonists (*e.g*., thrombin, serotonin (5-HT) or thromboxane A_2_) are exhibited by other arthropods (Additional file 1 Figure 1).

**Figure 1:**
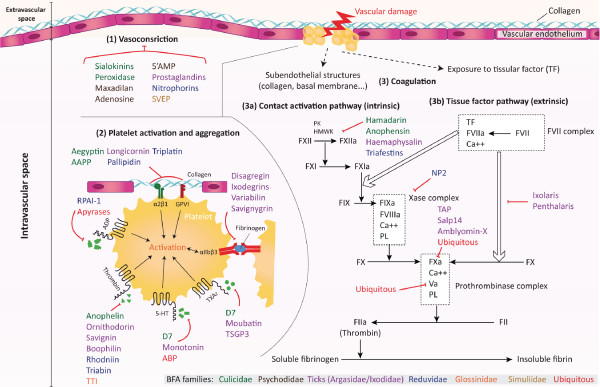
**Schematic representation of arthropod salivary proteins acting on primary and secondary haemostasis**. Haematophagous arthropods (HA) induce injuries to vascular endothelium when probing for a blood meal. The initial event of this vascular damage is vasoconstriction (1), which retards extravascular blood loss and enhances the adhesion of platelets to exposed subendothelial collagen. This adhesion activates platelets (2) and causes the release of platelet activation agonists (Adenosine diphosphate (ADP), Thrombin, Thromboxane A_2 _(TXA_2_), serotonin (5-HT)) as well as platelet membrane integrin receptor αIIbβ3. Fibrinogen binds to this receptor and crosslinks platelets to form a platelet plug. The blood coagulation cascade (3) is then initiated to strengthen the platelet plug with fibrin at the site of injury. The coagulation cascade is separated into two pathways converging into a common pathway. The contact activation pathway (intrinsic) involves high-molecular weight kininogen (HMWK), prekallikrein (PK), factor XII, factor XI and factor IX (3a), and the tissue factor pathway (extrinsic) involves the tissue factor and factor VII complex (3b). Both pathways lead to the activation of factor X. The common pathway leads to the generation of thrombin from prothrombin and the ultimate production of insoluble fibrin from fibrinogen. HA have evolved anti-haemostatic salivary proteins that inhibit specific agonists and factors of platelet aggregation and the blood coagulation cascade. The known actions of some HA salivary proteins listed in Additional file 1 are indicated. (Salivary protein affiliation to HA families is indicated by colour as represented on the bottom right corner legend).

This platelet activation prepares the implementation of secondary haemostasis by exposing the surface of activated platelets to coagulation proteins. Through a series of reactions involving several blood coagulation factors, the coagulation pathway (including the contact activation pathway and tissue factor pathway) is propagated until the formation of thrombin. The latter converts circulating soluble fibrinogen into insoluble fibrin, leading to blood clotting and complete cessation of haemorrhage [81-83]. As a result of convergent evolution, a variety of unrelated arthropod species have developed salivary inhibitors toward thrombin, a prime target to overcome both primary and secondary haemostasis (Additional file 1 Figure 1). As observed for primary haemostasis, more than one anticoagulant compound can be found in the saliva of a single haematophagous arthropod, as exemplified by hamadarin and anophensin, which are two anticoagulants targeting the contact activation pathway isolated in *An.stephensi *[84,85]. This redundancy of function reinforces the efficiency of the anti-haemostatic response.

### Salivary components and host's immunity

Adding to haemostatic defences, vertebrate hosts have evolved systems of immune defences to eliminate foreign organisms in the body, which can largely impair haematophagous arthropod blood-feeding. Tissue injury causes the immediate onset of acute inflammation and innate immunity, which promote tissue repair, prevent colonisation of the damaged tissues by opportunistic pathogens and initiates adaptive immunity, which is more specific.

Inflammation is characterised by multiple interactions between resident cells of the epidermis and dermis, such as endothelial cells, leucocytes, mast cells, neutrophils and platelets, which are the first to make contact with arthropod mouthparts as well as their saliva and their potential pathogens. These cells release pro-inflammatory mediators and chemotactic factors such as histamine, macrophage inflammatory protein-1α (MIP-1α) and leukotrienes [86-88], which activate and recruit leucocytes at the site of haemorrhage. The majority of salivary molecules inhibiting or scavenging these pro-inflammatory agonists were extensively studied in tick saliva (e.g., argasid or ixodid) compared to other arthropods (Additional file 1 Figure 2). Due to their habit of remaining attached to their host for a long period to feed until repletion, ticks are strongly dependent upon the potent immunosuppressive activities of their salivary components. Tick salivary components can then act on different actors of the innate immune response, such as the complement system [89-91], macrophages [92], natural killer cells [93,94] and the synthesis of proinflammatory cytokines [95,96], to succeed their blood meal. The suppressive effect induced by saliva on innate immunity was less studied in other haematophagous arthropods. Even though whole saliva or salivary gland extracts from Culicidae and Psychodidae have been found to induce a suppressive effect on innate immunity [97,98], several immunosuppressor components remain to be determined at the molecular level.

**Figure 2:**
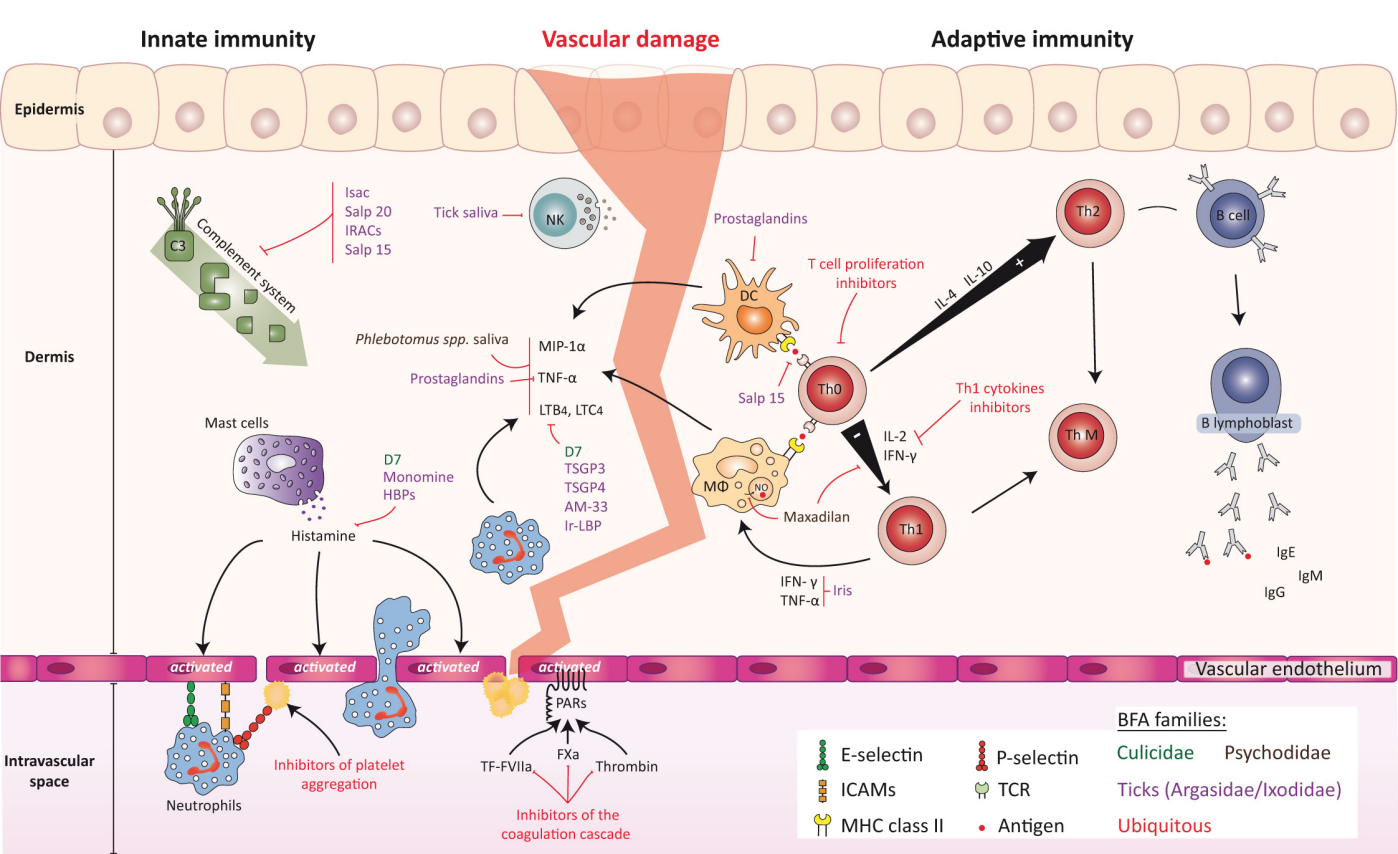
**Schematic representation of arthropod salivary proteins involved in the modulation of innate and adaptive immunity**. Protective immunity against haematophagous arthropods (HA) involves both innate and adaptive immunity. Cells involved in the innate response (*e.g*., neutrophils, natural killers cells (NK), mast cells and macrophages (MΦ)) represent the first line of defence. Once activated, these cells release molecules (*e.g*., macrophage inflammatory proteins -1 α (MIP-1α), tumour necrosis factor- α (TNF- α) or leukotrienes (LB_4_, LTC_4_) that initiate the inflammation process. This local inflammation can further be triggered by the activation of complement, which has chemotactic and inflammatory properties. Endothelial cells and platelets can be activated by the binding of factors of the coagulation cascade to PAR receptors, leading to an over-expression of surface adhesive molecules (ICAMs, E-selectin, P-selectin) that participate in neutrophil migration. Antigen presenting cells, such as dendritic cells (DC) migrate to the lymph nodes where they interact with naïve CD4+ helper T lymphocytes (Th0 cells) via the interplay of their T cell receptors (TCR) and major histocompatibility complex (MHC) class II proteins. Th0 cells have the potential to proliferate and to differentiate into two distinct lineages of effectors cells: Th1 and Th2 cells. Memory T helper (Th M) cells, which can improve the quality of the response to a subsequent exposure by developing more efficient memory capacity over time, are also produced. In a general pattern, HA saliva down-regulates the expression of Th1 cytokines (such as IL-2) modulating the adaptive immune response to an antibody mediated Th2 response. The action of saliva or salivary proteins is indicated in the figure as well as their corresponding organism's family. (Salivary protein affiliation to HA families is indicated by colour as represented on the bottom right corner legend).

Tissue damage and inflammation can lead to the extracellular production of adenosine from ATP degradation. Adenosine can have analgesic or pronociceptive effects depending on the activation of different peripheral receptors [99]. Pain perception can induce defensive behaviours from the host that could be deleterious to haematophagous arthropods. As a consequence, adenosine deaminase enzymes detected in saliva of various arthropods [100-102] were proposed to suppress pain perception by degrading adenosine at the bite site [101].

The innate immune system can also influence the type of adaptive immune response that develops. Haematophagous arthropod saliva can greatly impair the development of an appropriate adaptive immune response by the host by altering the function of antigen presenting cells (APC), such as macrophages [103] or dendritic cells (DC) [95,104,105]. These cells are involved in the capture and processing of salivary or pathogen antigens at the bite site, as well as in antigen presentation to T lymphocytes in the draining lymph nodes [106], which promotes cell and antibody mediated responses [107,108]. As a generalized pattern, salivary gland extracts from several haematophagous arthropods can inhibit Th1 cytokines secretion, such as IFN-γ and IL-2 [109-112], promoting the development of an antibody-mediated Th2 response [113-117]. This polarisation of host immunity toward a Th2 response (to the detriment of a Th1 cell-mediated response) is beneficial to the success of the blood feeding but it may also have a beneficial impact on pathogen transmission.

### Salivary components and enhancement of vector-borne pathogens infection

The discovery of the immuno-modulatory property of saliva has stimulated several research groups to study the involvement of salivary proteins from diverse vectors in the transmission and the establishment of corresponding pathogens into their hosts.

Titus and Ribeiro were the first to describe the enhancing effect of sand fly salivary gland extracts on cutaneous leishmaniasis when coinoculated with *Leishmania *promastigotes. Mice injected with *Leishmania *parasites concomitantly with a small amount of salivary gland proteins developed larger lesions and harboured more parasites than controls [118]. The enhancing effect of salivary extracts was confirmed with other *Leishmania *and sand fly species [119,120]. Subsequent studies have demonstrated that the injection of Triatominae bug saliva into the skin of mice in the presence of *Trypanosoma cruzi *parasites induced an up to six-fold blood parasitaemia [121], and that the saliva of ixodid ticks potentiated the transmission of Thogoto virus [122]. Interestingly, enhancement of Thogoto virus infection was only observed with salivary gland extracts derived from metastriate ixodid ticks but not from prostriate ixodid ticks, argasid ticks or mosquito saliva [122]. These results highlight the strong specificity of the vector/pathogen interaction that is needed in order to potentiate this enhancing transmission effect and suggest that this effect may involve a limited number of specific proteins to the haematophagous arthropod species or genus.

Effectively, in *Lutzomyia longipalpis *sand fly, the vasodilator maxadilan appears as the principal salivary molecule responsible for this enhanced parasite transmission as it exacerbates infection with *Leishmania major *to the same degree as whole saliva [123]. For *Borrelia burgdorferi*, the spirochetal agent of Lyme disease, the Salp15 protein expressed in *Ixodes scapularis *tick saliva enhances its transmission and survival within the vertebrate host. The spirochaete pathogen specifically up-regulates the expression of Salp15 and associates with it in order to be protected from borreliacidal effects induced by antibody-mediated killing [124]. The enhanced infection induced by saliva seems to be a widespread phenomenon in various vector species and for various viruses, bacterial or parasite pathogens [121,122,125,126]. Mosquito saliva might also accelerate and amplify infections of West Nile virus [127,128], La Crosse virus [129] or Cache-valley virus [130]. All these data suggest that haematophagous arthropod vectors are not simply "flying or crawling" syringes but rather play a dynamic role in the host/vector/pathogen relationship. The participation of saliva components in this transmission is supported by the increase infectivity observed when pathogens are delivered to the host by haematophagous arthropod bites compared to delivery by a syringe without saliva proteins [129,131-133].

All salivary proteins characterized in various hematophagous arthropods so far give a global overview of their complexity as well as their diversity both at their molecular level as well as their targets. It is interesting to note that only a minority of these salivary proteins has been assigned a precise function. For instance, concerning any tick species with a known genome or salivary gland transcriptome, less than 5% of the salivary proteins have their function verified [5]. Further knowledge on the pharmacology of arthropod salivary proteins might thus lead to the discovery of novel vasodilator, anti-platelet, anti-clotting, analgesic or immunomodulatory compounds, used by hematophagous arthropods to counteract host defenses. In addition, these salivary molecules might also provide new immunological tools to combat the direct and indirect nuisance caused by these hematophagous arthopods.

## Salivary proteins and host antibody response: immunological tools in sight?

### Saliva of haematophagous arthropods and allergy

Since the mid-1930s, numerous studies have described host immediate-type hypersensitivity (ITH) reactions in response to the bite of various haematophagous arthropod families, such as Psychodidae [134], Culicidae [135-137] or Glossinidae [138]. This ITH skin reaction, also known as type I hypersensitivity, is now widely accepted to be an allergic reaction which involves the production of IgE antibodies in response to specific salivary allergens [6]. Several studies have attempted to characterise the allergens involved in ITH by using different techniques (*e.g.*, skin testing, RAST, ELISA, Immunoblot) and different allergen preparations with the aim of developing tools for diagnosis and treatment of allergic reactions [139,140]. The comparison of the allergenic potency of whole body, thoracic and abdominal hemolymph and salivary glands from *Triatoma protracta *(reduviid bug) by RAST inhibition demonstrated that the allergens were concentrated in the salivary glands [139]. Similar results were observed for the *Ixodes holocyclus *tick [141]. Recently, Wongkamchai and colleagues have gained further evidence that major allergens are more abundant in saliva, followed by salivary gland extracts and whole body extracts from four mosquito species [142]. These different studies confirmed that the more concentrated source of allergens is located in saliva and salivary glands from haematophagous arthropods. Thus, saliva appears to be the prime antigenic source for testing or treating haematophagous arthropod-induced allergic reactions [143].

However, the collection of saliva is tedious, time consuming and constitutes a major drawback to the widespread medicinal use of these salivary components [143]. Hence, the synthetic production of arthropod vector saliva allergens is a promising alternative strategy for producing safe and highly standardized allergens on a large scale. A panel of studies using the immunoblot method have revealed a number of salivary proteins detected by IgE antibodies of individuals with skin hypersensitivity to arthropod bites, including mosquitoes [140,144-148], ticks [141,149] or reduviid bugs [139,150]. Some of these salivary allergens are now well characterised. For example, three recombinant *Aedes aegypti *salivary allergens corresponding to a 68 kDa salivary apyrase (rAed a1), a 37-kDa protein belonging to the D7 family (rAed a2) and a 30 kDa salivary gland allergen (rAed a3) elicit predominantly IgE responses in mosquito-allergic individuals [151,152]. The authors concluded that these recombinant allergens could greatly facilitate the diagnosis and immunotherapy of mosquito allergies. Recombinant salivary allergens were also evaluated in other haematophagous arthropod species and are presented in Table 2.

**Table 2:** Recombinant salivary proteins characterized in hematophagous arthropods and their immunological applications

Protein names	Organisms	Additional informations	MW [kDa]	Application	**Ref**.
rAed a1	*Aedes aegypti*	Salivary apyrase	68	Allergy	[151,152]
rAed a2	*Aedes aegypti*	Belong to the D7 family	37	Allergy	[151,152]
rAed a3	*Aedes aegypti*	30 kDa salivary gland allergen	30	Allergy	[151,152]
Procalin	*Triatoma protracta*	Belong to the lipocalin family	20	Allergy	[225]
Arg r 1	*Argas reflexus*	Belong to the lipocalin family	17	Allergy	[227]
Der-p2	*Ixodes ricinus*	*Dermatophagoides pteronyssinus *allergen- like	15.6	Allergy	[226]
TAg5	*Glosina m. morsitans*	Tsetse Antigen 5	28.9	Allergy	[228]
Maxadilan	*Lutzomyia longipalpis*	-	9.5	Vaccine candidate	[123]
SP15	*Phlebotomus papatasi*	-	15	Vaccine candidate	[162]
rLJM19	*Lutzomyia longipalpis*	-	11	Vaccine candidate	[229]
Salp15	*Ixodes scapularis*	-	14.7	Vaccine candidate	[163]
gSG6	*Anopheles gambiae*	-	10	Immunological marker of exposure	[218,230,220]
rTC	*Amblyomma. americanum*	Calreticulin	47.5	Immunological marker of exposure	[221]
rLJM11	*Lutzomyia longipalpis*	Yellow-related protein	43	Immunological marker of exposure	[223,224]
rLJM17	*Lutzomyia longipalpis*	Yellow-related protein	45	Immunological marker of exposure	[223,224]

Currently, whole body extracts from mosquitoes are used in the diagnosis and immunotherapy of mosquito bite allergies [153,154]. However, these commercially available samples contain many extraneous proteins that are not present in mosquito saliva and might interfere with diagnostics or may even cause additional sensitisation in subjects with a history of allergic reactions to mosquito bites. Moreover, the treatment of mosquito allergies is not widely used because considerable variations in the biological activity of these mosquito whole body allergen extracts have been described [155]. Thus, synthetic allergens appear to be a promising alternative for the diagnosis of allergic individuals, but also may improve desensitisation protocols and overcome the lack of standardisations in allergen immunotherapy [7,142].

### Saliva of haematophagous arthropods and vaccines

Over the course of the past 20 years, it has been observed that a history of exposure to uninfected bites has the ability to protect against several vector-borne infections, including tularaemia [156] and Lyme borreliosis [157] in animals pre-exposed to tick bites. The hypothesis that salivary components could be effective vaccine candidates for reducing the morbidity of vector-borne diseases in exposed individuals was strengthened by the discovery that pre-exposure of mice to salivary gland extracts of *Phlebotomus papatasii *abrogates the size of dermal lesions and reduces *Leishmania major *parasite loads in tissue [158]. More recently, it was shown that pre-exposing mice to *Anopheles stephensi *bites could protect them from rodent malaria [159], but these results are controversial [160].

These protective effects might be partly due to the development of host immunity against vector salivary proteins described as enhancing pathogen establishment. Based on these reflections, Morris and colleagues have tested the potential of *Lutzomia longipalpis *maxadilan as a vaccine candidate to protect mice against *Leishmania *infection (Table 2) [123]. Mice vaccinated with synthetic maxadilan were highly resistant to infection, as evidenced by smaller cutaneous lesions and a shorter healing period compared to controls. As maxadilan is only expressed in New World Psychodidae from the genus *Lutzomia *[68], it cannot confer protection against *Leishmania *infection transmitted by Old World sand flies from the genus *Phlebotomus*. However, vaccination with SP15, a 15 kD salivary protein from the Old World *P. papatasi *sand fly, protected mice from *Leishmania *infection [161,162]. These results highlight the challenge in developing a universal vaccine to control a specific pathogen transmitted by several vector species. Indeed, the variability in the salivary repertoire of closely related vector species implies that one must develop a salivary vaccine candidate for each different vector transmitting a specific pathogen. Additionally, one must take into account the geographical distribution of these vectors to determine the appropriate candidates that should be used during vaccination campains.

The efficacy of the Salp15 salivary protein as a vaccine candidate, the other well described salivary molecule isolated in *I. scapularis *ticks with an enhancing effect on pathogen transmission, was also tested *in vivo*. Mice immunised with recombinant Salp15 proteins were partially protected against Lyme borreliosis spirochetes transmitted by *I. scapularis *ticks [163]. Interestingly, the co-immunisation with Salp15 and OspA (a *Borrelia burgdorferi *outer-surface protein [164]) exerts a better protection against *B. burgdorferi *than either of these two candidates when used alone. Thus, the conjunction of salivary proteins to traditional pathogen-based vaccine could improve host protection against vector-borne disease infection. To our knowledge, no vaccine candidates have been developed against salivary components from other haematophagous arthropods. This relatively new vaccine approach (*i.e*., targeting arthropod salivary components required by a pathogen for its establishment in the host) necessitates the characterisation of salivary components exhibiting an enhancing effect on pathogen infection. The development of multi-epitope vaccines by the combination of pathogen-derived antigens with appropriate salivary antigens from their corresponding vectors could provide a better protection against vector-borne diseases than pathogen-derived vaccine candidates alone. Recently, *An. stephensi *saliva was reported to enhance the progression of cerebral malaria in a murine model [165]. The further characterisation of salivary components involved in this effect might lead to potential vaccine candidates, which could be used in combination with other malaria vaccine candidates to protect against severe malaria [166,167].

Other vaccine strategies using salivary proteins were undertaken in order to reduce host/vector contact by avoiding blood intake or diminishing the duration of the blood meal, particularly in ticks [168-170]. Additionally, vaccine candidates targeting gut or body haematophagous arthropod antigens were also developed to either disrupt the biology of the vectors or to block pathogen transmission. These approaches are promising to control vector-borne diseases but are beyond the scope of this review; supplementary details can be found in other works [171-175].

### Saliva of haematophagous arthropods and exposure markers

#### Relationship between anti-saliva IgG responses and haematophagous arthropod exposure

The absence of an antibody response against saliva from mosquitoes or Culicoides midges in the sera of children [176] or horses [177] living in Iceland (a country exempt from these two biting arthropods) and the appearance of an IgG antibody responses in animals or humans following exposure to haematophagous arthropod bites [144,177-179] are strong arguments suggesting that the acquisition of an antibody response against haematophagous arthropod saliva is exposure dependent.

The correlation between arthropod exposure and the level of anti-saliva IgG antibody was first evidenced using the sera from outdoor workers (in New Jersey, U.S.A.) who had been exposed to *Ixodes damini *ticks during their forestry activities [180]. Moreover, a significant decrease of the IgG anti-tick saliva levels was observed in the absence of tick exposure for several months (from October to January) [180]. From this point, several serological analyses demonstrated a relation between the density of diverse haematophagous arthropods and the level of antibody responses against their saliva. A kinetic analysis of the serological response against *Aedes communis *saliva from individuals living in Finnish Lapland indicated that seasonal exposure to mosquito bites elicited more intense antibody responses toward salivary antigens [181]. In a larger cohort, using sera from 1,059 Canadian blood donors sampled before and after the summer mosquito exposure peak, Peng and colleagues showed significant higher level of IgG antibody against *Aedes vexans *saliva after the summer peak exposure [182]. Higher levels of anti-saliva IgG antibodies were also detected in individuals exposed to *Glossina *bites compared to non-exposed individuals [50]. These works demonstrated that levels of serological immune responses could be influenced by seasonal variations of the level of haematophagous arthropod densities.

Additionally, Orlandi-Pradines and colleagues have evaluated the consequences of a transient exposure to *An. gambiae *and *Ae. aegypti *mosquitoes in French travellers during a five-month journey to tropical Africa on anti-saliva IgG responses [49]. This study reported that several travellers from areas free of *An. gambiae *and *Ae. aegypti *mosquitoes developed an antibody response against saliva from these two unrelated mosquitoes. Thus, transient exposure (*e.g.*, seasons or travel into endemic areas) to haematophagous arthropod bites seems sufficient for developing an IgG response against arthropod saliva. Additionally, IgM antibodies directed against *Triatoma infestans *saliva can be detectable as early as one day after a single encounter with several triatomine bugs and decrease even more rapidly (18 days) than IgG in chickens [183]. These IgM responses seem highly sensitive to the detection of bug exposures; however, no association was observed between level of exposure and IgM antibody levels. These results highlight the potential use of the short persistence of IgM responses (as a complement to measuring IgG responses) as an indicator of recent exposure to haematophagous arthropods. The observed link between anti-saliva antibody responses and haematophagous arthropod exposure, as well as the waning of these antibody responses after a period of non-exposure, favour the potential use of immunogenic saliva as an immunological marker of exposure. Indeed, simple blood sampling would give an indication of individual exposure to the bites of specific vectors and could be used to complete entomological surveys or to replace them when human landing catch or other trapping methods are difficult to implement [184]. Saliva-based immunological markers of exposures would also be more appropriate than measuring vector-borne disease incidence in clinical trials to assess the effectiveness of anti-vectorial devices in areas with low pathogen transmission intensity [185]. Finally, it could be an alternative strategy compared to entomological methods (*i.e*., human landing catch) to assess vector bite exposure particularly in children [186].

In a recent clinical assay, Drame and colleagues confirmed the validity of using *An. gambiae *crude saliva as an immunological marker to assess the efficaccy of insecticide-treated nets (ITNs) in a malaria hypo-endemic transmission area [187]. They measured anti-saliva IgG levels, blood parasitaemia and vector densities before and after the introduction of ITNs. A significant decrease in the anti-saliva IgG response was observed after the introduction of ITNs. This diminution of antibody response was associated with a drop in parasite load but not with vector densities as measured by light traps, a standard but highly biased and imprecise entomological methods. Recently, antibody responses from sentinel guinea pigs to salivary proteins of *T. infestans*, the vector of *T. cruzi*, was shown to be a powerful tool for the evaluation of vector control interventions against Chagas disease [188]. These studies demonstrated that anti-saliva antibody responses could be efficient tools to assess the effectiveness of antivectorial strategies implemented to control diverse vector borne diseases by giving an estimation of the real intensity of haematophagous arthropod bites at the individual level. Thus, variations of the IgG antibody level appeared to be correlated with haematophagous arthropod density, which was dependent on several factors, such as seasons, ecological environments, individual activities or the level of anti-vectorial protection.

#### Diversity and specificity of salivary components

As some areas can exhibit a high biodiversity in terms of haematophagous arthropod species [189,190], a high level of specificity is necessary to assess individual exposure by immunological tests based on haematophagous arthropod saliva. Several studies have reported diverse degrees of cross-reactivity between different vector species, ranging from low [191,192] to high species-specificity [150,193-195]. The presence of cross-reactivity was often described in related species [191,192], suggesting that this phenomenon can occur in closely related saliva components.

**Figure 3:**
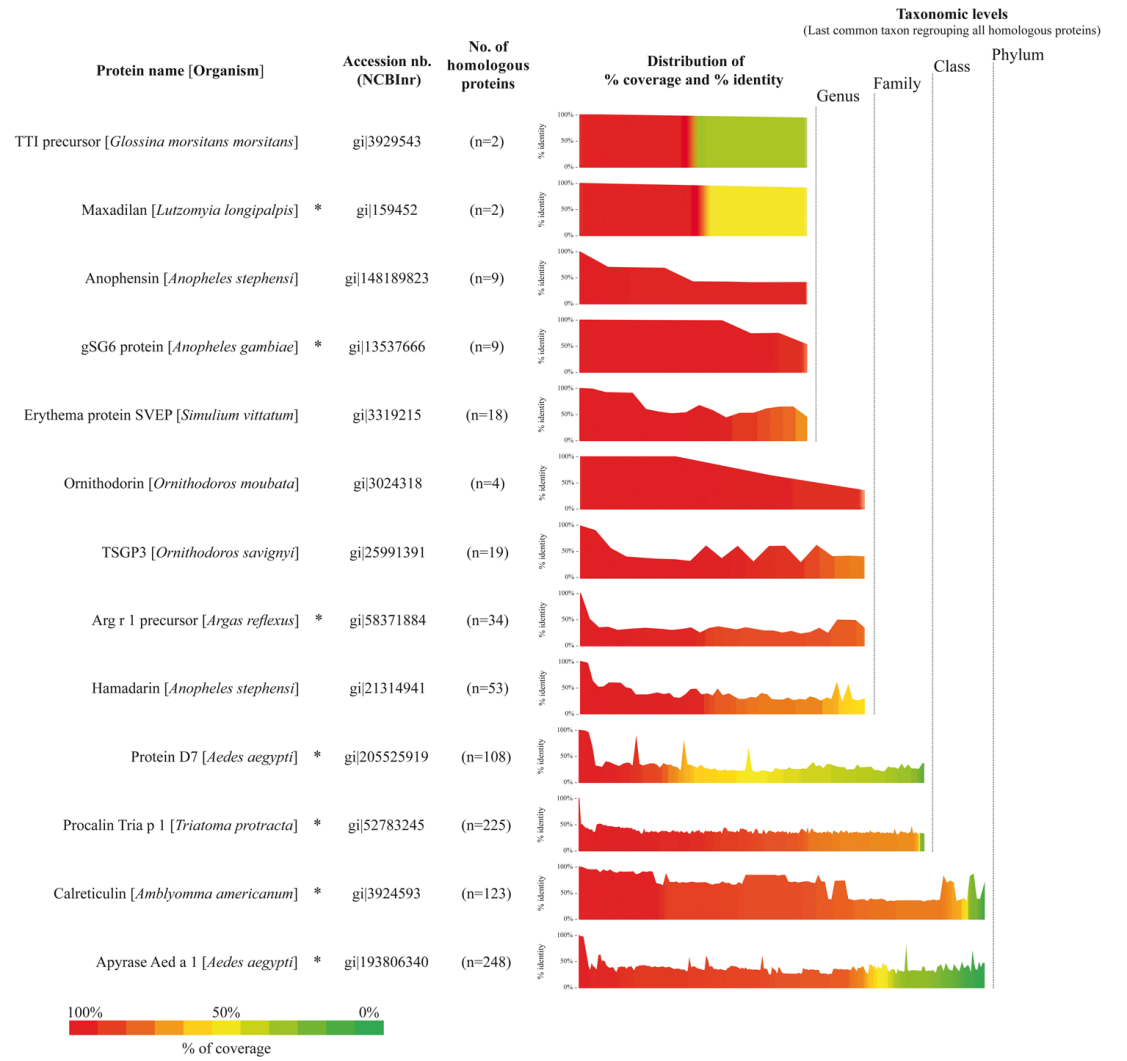
**Protein sequence diversity of haematophagous arthropods salivary proteins**. Sequences of 13 salivary proteins of haematophagous arthropods described in the present review were submitted to BLAST analysis on the non-redundant protein database (NCBInr, NIH, Bethesda). The blastp program was used with default parameters excepting the following: search was done on the *Arthropoda *taxonomic level (taxid: 6656, Nov 15^th^, 2010, 12,289,957 sequences), the E-value threshold was changed to a setting of 1 in order to recover only hits with highest significance on the overall protein sequence and the hit-list size was set to 5000 proteins. The number of homologous proteins with a score above 40 and their respective percentage of coverage and identity were recovered for all query proteins and sorted according to their increasing percentage of coverage. The number of homologous proteins is indicated in brackets (this includes the query sequence) and the distributions of both the percentage of coverage (bar graph with a coloured scale) and the percentage of identity (line profile above each bar graph) are represented. Proteins are grouped according to the taxonomic level of the last common taxon regrouping their corresponding homologous proteins. For graphical convenience, subclass, class and infraclass as well as superfamily, family and subfamily taxonomic levels were grouped into class and family, respectively. Salivary proteins reported to be targeted by an immune response are indicated by an asterisk (*).

The specificity of the saliva based immunological test is a prerequisite to assess individual exposure to a specific genus or species of arthropods. An important step forward in the knowledge of the salivary protein diversity in the phylum Arthropoda was the cataloguing of salivary gland proteins expressed and secreted in several species of haematophagous arthropods. The recent elucidation of the genome of major haematophagous arthropods [196-200] added to increasing transcriptomic and proteomic work on salivary glands, making it possible to identify salivary molecules in various haematophagous arthropods. To date, the transcription repertoire (named sialotranscriptome) of at least 30 different species of haematophagous arthropods has been drawn up, revealing a number of both ubiquitous and specific proteins throughout the taxonomic hierarchy [201-208]. The independent evolution of haematophagous arthropods and host immune pressure over the salivary products led to a diversity of pharmacological molecules even among different genera within a same family [205,209]. An insight into the taxonomic variability at the protein sequence level of some haematophagous arthropod salivary proteins is shown on Figure 3. The homology/diversity of salivary proteins can be observed at two levels: (i) Homologous salivary proteins can be conserved at different taxonomic levels from genus to the entire arthropod phylum and (ii) the distribution of percentage identity of homologous proteins inside each taxonomic level is highly variable. This supports the existence of numerous candidates that can be used to assess individual exposure to specific haematophagous arthropods.

Interestingly, studies on haematophagous arthropod saliva generally use inbred laboratory strains. However, for one species, there exist distinct colonies coming from arthropods collected in the field that differed in their origins and laboratory colonisation histories. In order to evaluate sialome divergence, which could occur following the rearing of haematophagous arthropods over several decades (*e.g.*, mosquitoes), under laboratory conditions, the sialomes (*i.e.*, saliva and salivary gland) of three mosquito colonies (*i.e., Ae. aegypti *colonies *Rockefeller, PAEA *and *Formosus*) were compared using 1D SDS-PAGE [143,210]. At the saliva and salivary gland level, no major differences were detected between these three colonies, suggesting that the expression of salivary proteins is highly conserved across populations. But these data could not exclude the possibility that a long history of laboratory rearing might have induced a homogenisation of salivary protein repertoires, which may differ from their field counterparts. This loss of salivary protein diversity as a result of long-term colonization was hypothetised to be responsible of the observed positive effects on the outcome of *Leishmania *infection on mice pre-immunized with sand fly saliva [211]. Indeed, pre-immunization of mice with saliva from long-term colonized phlebotomine sand flies, was reported to induce a better protection against *Leishmania *infection than saliva from wild-caught or recently colonised sand flies [211,212]. This preserved repertoire of salivary proteins at the species level is essential to develop anti-saliva based immunological tools to assess individual exposure to different haematophagous arthropod colonies settled in various areas throughout the world. The access of salivary samples from wild-caught arthropods using convenient procedures adapted to field works would allow to assess the sialome diversity between laboratory reared and wild arthropods [213].

#### Synthetic salivary components as immunological markers of exposure

Major limits for developing a biological test of exposure to haematophagous arthropod bites are the difficulty of collecting saliva or salivary gland extracts and the lack of standardisation in sampling. Effectively, the salivary protein content of haematophagous arthropods can vary according to their sex, age or diets [214-216]. Thus, a gain of sensitivity, specificity and reproducibility could be obtained by identifying the genus or species-specific immunogenic salivary proteins and to produce them in recombinant form or synthetic peptides.

The recombinant *Anopheles gambiae *salivary gland protein 6 (gSG6), a small salivary protein highly preserved in the *Anopheles *genus [217], was evaluated as an immunological marker of exposure [218]. The recombinant protein was detected by IgG antibodies from children exposed to the bite of *An. gambiae*. BLAST analysis (NCBInr, NIH, Bethesda, Nov 15th, 2010) revealed that homologous proteins of gSG6 protein can only be found in 8 *Anopheles *species so far, suggesting its specificity to the *Anopheles *genus (Figure 3). This protein has recently been proposed as a serological candidate marker of exposure to Afrotropical malaria vectors [219,220]. Human hosts exposed to *Amblyomma americanum *and *Dermacentor variabilis *ticks also develop a specific IgG response against a recombinant calreticulin (rTC) protein isolated from the salivary glands of the *A. americanum *tick [221]. The use of recombinant salivary proteins, which are highly preserved between several related vector species, could be useful in assessing the risk of disease transmission in individuals living in areas where vector diversities are not well characterised at the species level. Interestingly, anti-rTC antibody seropositivity has higher specificity but lower sensitivity than antibodies directed against whole saliva in detecting individuals that have been exposed to ticks [222]. The use of a single recombinant salivary protein to assess individual exposure to tick bites may explain this lack of sensitivity. Indeed, the use of two recombinant proteins (named LJM17 and LJM11) was reported to be more effective and sensitive than whole saliva to estimate the level of exposure to *Lutzomia longipalpis *sand flies, vectors of *Leishmania *parasites [223,224].

Recombinant proteins that have been primarily produced for their biological properties or their role in allergic responses (Table 2) could also be considered potential markers of exposure candidates [225-229]. Some of these proteins appear to be relatively specific to the vector family or genus (Figure 3) and might be promising epidemiological markers of vector exposure. Ribeiro and colleagues classified conserved salivary proteins at different taxonomic levels in the suborder Nematocera from which some other specific antigenic candidates might emerge [205].

The detection and selection of highly specific peptides inside the whole salivary protein sequence could further increase the specificity of such immunological markers and reduce production costs. In order to optimise the specificity of the gSG6 biomarker, Poinsignon and colleagues have designed a gSG6-based peptide sequence (gSG6-P1) according to its predicted immunogenic properties [218]. A positive association between the anti-gSG6-P1 IgG responses and the level of exposure was observed in individuals exposed to *An. gambiae *bites. This peptide was also detected in individuals exposed to a very low number of the malaria vector bites, suggesting its potential to reveal *An. gambiae *exposure in a context where classical entomological methods would be employed with difficulty (*i.e.*, urban areas, altitude, travellers) [230].

Taken together, all these data support the use of immunogenic salivary components as new tools for identifying individuals at risk to vector-borne diseases and for monitoring haematophagous arthropod populations and anti-vector intervention strategies. A gain of sensitivity and specificity could be achieved by the selection and production of recombinant antigens or peptides that do not share sequence homology with other haematophagous arthropod species. Such synthetic products increase the amount of available protein for large cohort studies using high-throughput methods such as Luminex technology. These multiplex assays are cost and time effective and have proven to be useful strategies for the detection of serum antibodies directed against infectious pathogens [231,232] and for evaluating individual exposure to vector borne diseases [233].

## Conclusion

In order to facilitate their blood meals, haematophagous arthropods have elaborated a wide range of salivary components that have essential roles in counteracting host haemostatic defences. In addition to these pharmacological activities, salivary components can modulate host immunity at the bite site and induce an immune environment favourable for pathogen transmission. This immuno-modulation is associated with the production of specific antibody responses. Since the 1980s, several studies have investigated the antibody responses of vertebrate hosts against salivary proteins in an initial attempt to treat uncomfortable allergic reactions to haematophagous arthropod bites. The immunogenic properties of some salivary proteins can be used as vaccine candidates for improving host protection against some vector-borne diseases. Salivary proteins are likely to become immunological markers for relevant estimation of vector/host contacts, of the effectiveness of various control or surveillance programs, and an estimation of the pathogen transmission risk to complement methods that are currently available. A gain in sensitivity, specificity and reproducibility is expected to be obtained by the identification of species-specific immunogenic salivary peptides or the combination of several recombinant salivary proteins.

## Competing interests

The authors declare that they have no competing interests.

## Authors' contributions

FA and AL conceived the intellectual content of the article. FA and AL collected results presented here and wrote first draft of the manuscript. FA designed all figures. FA, ID, NB NB, DM, FP, TF, CR and AL participated in the formation of the final version of manuscript. All authors read and approved the final manuscript.

## Supplementary Material

Additional file 1 **Anti-hemostatic and immunomodulatory salivary proteins in hematophagous arthropods**. Known anti-hemostatic and immunomodulatory properties of salivary proteins from diverse hematophagous arthropods are presented. Click here for file
