# Supplementary material for: Implication of haematophagous arthropod salivary proteins in host-vector interactions
Source: Parasit Vectors. 2011 Sep 28;4:187. doi: 10.1186/1756-3305-4-187 (PMC3197560; doi:10.1186/1756-3305-4-187)
Supplement: Additional file 1 — Anti-hemostatic and immunomodulatory salivary proteins in hematophagous arthropods. Known anti-hemostatic and immunomodulatory properties of salivary proteins from diverse hematophagous arthropods are presented. [file 1756-3305-4-187-S1.DOC]

Additional file 1: Anti-hemostatic and immunomodulatory salivary proteins in hematophagous arthropods

|  | **Arthropod families** | **Arthropod species** | **Protein names** | **Anti-hemostatic properties** | **Immunomodulatory properties** | **Ref.** |
| --- | --- | --- | --- | --- | --- | --- |
|  |  | *A. monolakensis* | Monotonin | inhibitor of platelet aggregation |  |  |
|  |  |  | Monomine |  | Anti-inflammatory |  |
|  |  |  | AM-33 |  | Anti-inflammatory |  |
|  |  | *O. moubata* | Tick anticoagulant peptide (TAP) | anticoagulant |  |  |
|  |  |  | Ornithodorin | inhibitors of platelet aggregation/ anticoagulant |  |  |
|  |  | *O. savignyi* | Savignygrin | inhibitor of plateletaggregation |  |  |
|  |  |  | Savignin | inhibitors of platelet aggregation/ anticoagulant |  |  |
|  |  |  | TSGP4 |  | Anti-inflammatory |  |
|  |  | Ubiq. | Moubatin | inhibitor of platelet aggregation |  |  |
|  |  |  | TSGP3 | inhibitor of platelet aggregation | Anti-inflammatory/anticomplement activity |  |
|  | Ixodidae | *Am. cajennense* | Amblyomin-X | anticoagulant |  |  |
|  |  | *B. microplus* | Boophilin | inhibitors of platelet aggregation/anticoagulant |  |  |
|  |  | *D. variabilis* | Variabilin | inhibitor of platelet aggregation |  |  |
|  |  | *D. reticulatus* | Uncharacterized salivary molecules |  | Decrease natural killer cells activity |  |
|  |  | *H. longicornis* | Haemaphysalin | anticoagulant |  |  |
|  |  |  | Longicornin | inhibitor of platelet aggregation |  |  |
|  |  |  |  |  |  |  |
|  |  | *I. pacificus* | Ixodegrins | inhibitor of platelet aggregation |  |  |
|  |  | *I. ricinus* | Ir-LBP |  | Anti-inflammatory |  |
|  |  |  | *I. ricinus* immunosuppressor (Iris) |  | Anti-inflammatory/T cell modulatory effect |  |
|  |  |  | *I. ricinus* anti-complement proteins (IRACs) |  | Anticomplement activity |  |
|  |  | *I. scapularis* | Ixolaris | anticoagulant |  |  |
|  |  |  | Salp14 | anticoagulant |  |  |
|  |  |  | Penthalaris | anticoagulant |  |  |
|  |  |  | *I. scapularis* salivary anti-complement (Isac) |  | Anticomplement activity |  |
|  |  |  | Salp20 |  | Anticomplement activity |  |
|  |  | *I. spp.* | Salp15 |  | Anticomplement activity/T cell modulatory effect |  |
|  |  | *O. moubata* | Disagregin | inhibitor of platelet aggregation |  |  |
|  |  | *R. appendiculatus* | histamine-bindingproteins (HBPs) |  | Anti-inflammatory |  |
|  | Ixodidoidae | Ubiq. | Prostaglandins | vasodilator | Anti-inflammatory/inhibitor of DC maturation |  |
|  | Psychodidae | *Lu.longipalpis* | Maxadilan | vasodilator | T cell modulatory effect |  |
|  |  | *Ph. papatasi* | Adenosine | vasodilator/inhibitors of platelet aggregation |  |  |
|  |  |  | 5’AMP | vasodilator/inhibitor of platelet aggregation |  |  |
|  |  | *Phlebotomus spp.* | Neutrophil chemotactic mediators inhibitors |  | Anti-inflammatory |  |
|  | Simuliidae | *S. vittatum* | *S. vittatum* erythema protein (SVEP) | vasodilator |  |  |
| Capillary feeders | Culicidae | *Ae. aegypti* | Aegyptin | inhibitor of plateletaggregation |  |  |
|  |  |  | Sialokinins | vasodilator | T cell modulatory effect |  |
|  |  | *An. albimanus* | Peroxidase | vasodilator |  |  |
|  |  |  | Anophelin | inhibitors of platelet aggregation/ anticoagulant |  |  |
|  |  | *An. stephensi* | Hamadarin | anticoagulant |  |  |
|  |  |  | Anophensin | anticoagulant |  |  |
|  |  |  | Anopheline antiplatelet protein (AAPP) | inhibitor of plateletaggregation |  |  |
|  |  | Ubiq. | D7 | vasodilator/inhibitor of platelet aggregation | Anti-inflammatory |  |
|  | Glossinidae | *G. m. morsitans* | Tsetse thrombin inhibitor (TTI) | Inhibitor of platelet aggregation/ anticoagulant |  |  |
|  | Reduviidae | *Rh. prolixus* | Rhodniin | inhibitor of platelet aggregation/ anticoagulant |  |  |
|  |  |  | *R. prolixus* aggregation inhibitor 1 (RPAI-1) | inhibitor of platelet aggregation |  |  |
|  |  | *T. infestans* | Triafestins | anticoagulant |  |  |
|  |  |  | Triplatin | inhibitor of platelet aggregation |  |  |
|  |  | *T. pallidipennis* | Pallidipin | inhibitor of platelet aggregation |  |  |
|  |  |  | Triabin | inhibitors of platelet aggregation/ anticoagulant |  |  |
|  |  | Ubiq. | Nitrophorins | vasodilator/ anticoagulant |  |  |
| Ubiquitous | Ubiquitous | - | Amine-bindingproteins (ABP) | vasodilator/inhibitor of platelet aggregation |  |  |
|  |  | - | Apyrase | inhibitor of platelet aggregation |  |  |
|  |  | - | Uncharacterized FXa-directed anticoagulants | anticoagulant |  |  |
|  |  | - | Uncharacterized FV-directed anticoagulants | anticoagulant |  |  |
|  |  | - | Uncharacterized immunomodulatory molecules |  | T cell modulatory effect |  |

*O.*: *Ornithodoros*; *I.*: *Ixodes* ; *A.* : *Argas*, *Am.* : *Amblyomma*, *H.*: *Haemaphysalis* ; *D.*: *Dermacentor*; *B.* : *Boophilus* ; *R.* : *Rhipicephalus*; *Lu.*: *Lutzomia ; Ph.*: *Phlebotomus*; *S.*: *Simulium*; *An.*: *Anopheles ; Ae.*: *Aedes*; *G.*: *Glossina*; *T.*: *Triatoma* ; *Rh.*: *Rhodnius*; Ubiq. : Ubiquitous. The term ubiquitous was used when a salivary protein was described in more than one arthropod species or family.

**References :**

1. Mans BJ, Ribeiro JM, Andersen JF: **Structure, function, and evolution of biogenic amine-binding proteins in soft ticks.** *J Biol Chem* 2008, **283:**18721-18733.

2. Mans BJ, Ribeiro JM: **A novel clade of cysteinyl leukotriene scavengers in soft ticks.** *Insect Biochem Mol Biol* 2008, **38:**862-870.

3. Waxman L, Smith DE, Arcuri KE, Vlasuk GP: **Tick anticoagulant peptide (TAP) is a novel inhibitor of blood coagulation factor Xa.** *Science* 1990, **248:**593-596.

4. van de Locht A, Stubbs MT, Bode W, Friedrich T, Bollschweiler C, Hoffken W, Huber R: **The ornithodorin-thrombin crystal structure, a key to the TAP enigma?** *EMBO J* 1996, **15:**6011-6017.

5. Mans BJ, Louw AI, Neitz AW: **Savignygrin, a platelet aggregation inhibitor from the soft tick *Ornithodoros savignyi*, presents the RGD integrin recognition motif on the Kunitz-BPTI fold.** *J Biol Chem* 2002, **277:**21371-21378.

6. Mans BJ, Louw AI, Neitz AW: **Amino acid sequence and structure modeling of savignin, a thrombin inhibitor from the tick, *Ornithodoros savignyi*.** *Insect Biochem Mol Biol* 2002, **32:**821-828.

7. Mans BJ, Ribeiro JM: **Function, mechanism and evolution of the moubatin-clade of soft tick lipocalins.** *Insect Biochem Mol Biol* 2008, **38:**841-852.

8. Batista IF, Ramos OH, Ventura JS, Junqueira-de-Azevedo IL, Ho PL, Chudzinski-Tavassi AM: **A new Factor Xa inhibitor from *Amblyomma cajennense* with a unique domain composition.** *Arch Biochem Biophys* 2010, **493:**151-156.

9. Macedo-Ribeiro S, Almeida C, Calisto BM, Friedrich T, Mentele R, Sturzebecher J, Fuentes-Prior P, Pereira PJ: **Isolation, cloning and structural characterisation of boophilin, a multifunctional Kunitz-type proteinase inhibitor from the cattle tick.** *PLoS One* 2008, **3:**e1624.

10. Wang X, Coons LB, Taylor DB, Stevens SE, Jr., Gartner TK: **Variabilin, a novel RGD-containing antagonist of glycoprotein IIb-IIIa and platelet aggregation inhibitor from the hard tick *Dermacentor variabilis*.** *J Biol Chem* 1996, **271:**17785-17790.

11. Kubes M, Kocakova P, Slovak M, Slavikova M, Fuchsberger N, Nuttall PA: **Heterogeneity in the effect of different ixodid tick species on human natural killer cell activity.** *Parasite Immunol* 2002, **24:**23-28.

12. Kato N, Iwanaga S, Okayama T, Isawa H, Yuda M, Chinzei Y: **Identification and characterization of the plasma kallikrein-kinin system inhibitor, haemaphysalin, from hard tick, *Haemaphysalis longicornis*.** *Thromb Haemost* 2005, **93:**359-367.

13. Cheng Y, Wu H, Li D: **An inhibitor selective for collagen-stimulated platelet aggregation from the salivary glands of hard tick *Haemaphysalis longicornis* and its mechanism of action.** *Sci China C Life Sci* 1999, **42:**457-464.

14. Francischetti IM, My Pham V, Mans BJ, Andersen JF, Mather TN, Lane RS, Ribeiro JM: **The transcriptome of the salivary glands of the female western black-legged tick *Ixodes pacificus* (Acari: Ixodidae).** *Insect Biochem Mol Biol* 2005, **35:**1142-1161.

15. Beaufays J, Adam B, Menten-Dedoyart C, Fievez L, Grosjean A, Decrem Y, Prevot PP, Santini S, Brasseur R, Brossard M, et al: **Ir-LBP, an *Ixodes ricinus* tick salivary LTB4-binding lipocalin, interferes with host neutrophil function.** *PLoS One* 2008, **3:**e3987.

16. Leboulle G, Crippa M, Decrem Y, Mejri N, Brossard M, Bollen A, Godfroid E: **Characterization of a novel salivary immunosuppressive protein from *Ixodes ricinus* ticks.** *J Biol Chem* 2002, **277:**10083-10089.

17. Daix V, Schroeder H, Praet N, Georgin JP, Chiappino I, Gillet L, de Fays K, Decrem Y, Leboulle G, Godfroid E, et al: **Ixodes ticks belonging to the *Ixodes ricinus* complex encode a family of anticomplement proteins.** *Insect Mol Biol* 2007, **16:**155-166.

18. Nazareth RA, Tomaz LS, Ortiz-Costa S, Atella GC, Ribeiro JM, Francischetti IM, Monteiro RQ: **Antithrombotic properties of Ixolaris, a potent inhibitor of the extrinsic pathway of the coagulation cascade.** *Thromb Haemost* 2006, **96:**7-13.

19. Narasimhan S, Koski RA, Beaulieu B, Anderson JF, Ramamoorthi N, Kantor F, Cappello M, Fikrig E: **A novel family of anticoagulants from the saliva of *Ixodes scapularis*.** *Insect Mol Biol* 2002, **11:**641-650.

20. Francischetti IM, Mather TN, Ribeiro JM: **Penthalaris, a novel recombinant five-Kunitz tissue factor pathway inhibitor (TFPI) from the salivary gland of the tick vector of Lyme disease, *Ixodes scapularis*.** *Thromb Haemost* 2004, **91:**886-898.

21. Valenzuela JG, Charlab R, Mather TN, Ribeiro JM: **Purification, cloning, and expression of a novel salivary anticomplement protein from the tick, *Ixodes scapularis*.** *J Biol Chem* 2000, **275:**18717-18723.

22. Tyson K, Elkins C, Patterson H, Fikrig E, de Silva A: **Biochemical and functional characterization of Salp20, an *Ixodes scapularis* tick salivary protein that inhibits the complement pathway.** *Insect Mol Biol* 2007, **16:**469-479.

23. Schuijt TJ, Hovius JW, van Burgel ND, Ramamoorthi N, Fikrig E, van Dam AP: **The tick salivary protein Salp15 inhibits the killing of serum-sensitive *Borrelia burgdorferi* sensu lato isolates.** *Infect Immun* 2008, **76:**2888-2894.

24. Juncadella IJ, Garg R, Ananthnarayanan SK, Yengo CM, Anguita J: **T-cell signaling pathways inhibited by the tick saliva immunosuppressor, Salp15.** *FEMS Immunol Med Microbiol* 2007, **49:**433-438.

25. Karczewski J, Endris R, Connolly TM: **Disagregin is a fibrinogen receptor antagonist lacking the Arg-Gly-Asp sequence from the tick, *Ornithodoros moubata*.** *J Biol Chem* 1994, **269:**6702-6708.

26. Paesen GC, Adams PL, Harlos K, Nuttall PA, Stuart DI: **Tick histamine-binding proteins: isolation, cloning, and three-dimensional structure.** *Mol Cell* 1999, **3:**661-671.

27. Bowman AS, Dillwith JW, Sauer JR: **Tick salivary prostaglandins: Presence, origin and significance.** *Parasitol Today* 1996, **12:**388-396.

28. Sa-Nunes A, Bafica A, Lucas DA, Conrads TP, Veenstra TD, Andersen JF, Mather TN, Ribeiro JM, Francischetti IM: **Prostaglandin E2 is a major inhibitor of dendritic cell maturation and function in *Ixodes scapularis* saliva.** *J Immunol* 2007, **179:**1497-1505.

29. Jackson TS, Lerner E, Weisbrod RM, Tajima M, Loscalzo J, Keaney JF, Jr.: **Vasodilatory properties of recombinant maxadilan.** *Am J Physiol* 1996, **271:**H924-930.

30. Brodie TM, Smith MC, Morris RV, Titus RG: **Immunomodulatory effects of the *Lutzomyia longipalpis* salivary gland protein maxadilan on mouse macrophages.** *Infect Immun* 2007, **75:**2359-2365.

31. Uchida D, Tatsuno I, Tanaka T, Hirai A, Saito Y, Moro O, Tajima M: **Maxadilan is a specific agonist and its deleted peptide (M65) is a specific antagonist for PACAP type 1 receptor.** *Ann N Y Acad Sci* 1998, **865:**253-258.

32. Qureshi AA, Asahina A, Ohnuma M, Tajima M, Granstein RD, Lerner EA: **Immunomodulatory properties of maxadilan, the vasodilator peptide from sand fly salivary gland extracts.** *Am J Trop Med Hyg* 1996, **54:**665-671.

33. Ribeiro JM, Katz O, Pannell LK, Waitumbi J, Warburg A: **Salivary glands of the sand fly *Phlebotomus papatasi* contain pharmacologically active amounts of adenosine and 5'-AMP.** *J Exp Biol* 1999, **202:**1551-1559.

34. Carregaro V, Valenzuela JG, Cunha TM, Verri WA, Jr., Grespan R, Matsumura G, Ribeiro JM, Elnaiem DE, Silva JS, Cunha FQ: **Phlebotomine salivas inhibit immune inflammation-induced neutrophil migration via an autocrine DC-derived PGE2/IL-10 sequential pathway.** *J Leukoc Biol* 2008, **84:**104-114.

35. Cupp MS, Ribeiro JM, Champagne DE, Cupp EW: **Analyses of cDNA and recombinant protein for a potent vasoactive protein in saliva of a blood-feeding black fly, *Simulium vittatum*.** *J Exp Biol* 1998, **201:**1553-1561.

36. Calvo E, Tokumasu F, Marinotti O, Villeval JL, Ribeiro JM, Francischetti IM: **Aegyptin, a novel mosquito salivary gland protein, specifically binds to collagen and prevents its interaction with platelet glycoprotein VI, integrin alpha2beta1, and von Willebrand factor.** *J Biol Chem* 2007, **282:**26928-26938.

37. Champagne DE, Ribeiro JM: **Sialokinin I and II: vasodilatory tachykinins from the yellow fever mosquito *Aedes aegypti*.** *Proc Natl Acad Sci U S A* 1994, **91:**138-142.

38. Zeidner NS, Higgs S, Happ CM, Beaty BJ, Miller BR: **Mosquito feeding modulates Th1 and Th2 cytokines in flavivirus susceptible mice: an effect mimicked by injection of sialokinins, but not demonstrated in flavivirus resistant mice.** *Parasite Immunol* 1999, **21:**35-44.

39. Ribeiro JM, Nussenzveig RH: **The salivary catechol oxidase/peroxidase activities of the mosquito *Anopheles albimanus*.** *J Exp Biol* 1993, **179:**273-287.

40. Francischetti IM, Valenzuela JG, Ribeiro JM: **Anophelin: kinetics and mechanism of thrombin inhibition.** *Biochemistry* 1999, **38:**16678-16685.

41. Isawa H, Yuda M, Orito Y, Chinzei Y: **A mosquito salivary protein inhibits activation of the plasma contact system by binding to factor XII and high molecular weight kininogen.** *J Biol Chem* 2002, **277:**27651-27658.

42. Isawa H, Orito Y, Iwanaga S, Jingushi N, Morita A, Chinzei Y, Yuda M: **Identification and characterization of a new kallikrein-kinin system inhibitor from the salivary glands of the malaria vector mosquito *Anopheles stephensi*.** *Insect Biochem Mol Biol* 2007, **37:**466-477.

43. Yoshida S, Sudo T, Niimi M, Tao L, Sun B, Kambayashi J, Watanabe H, Luo E, Matsuoka H: **Inhibition of collagen-induced platelet aggregation by anopheline antiplatelet protein, a saliva protein from a malaria vector mosquito.** *Blood* 2008, **111:**2007-2014.

44. Calvo E, Mans BJ, Andersen JF, Ribeiro JM: **Function and evolution of a mosquito salivary protein family.** *J Biol Chem* 2006, **281:**1935-1942.

45. Alvarenga PH, Francischetti IM, Calvo E, Sa-Nunes A, Ribeiro JM, Andersen JF: **The function and three-dimensional structure of a thromboxane A2/cysteinyl leukotriene-binding protein from the saliva of a mosquito vector of the malaria parasite.** *PLoS Biol* 2010, **8:**e1000547.

46. Cappello M, Li S, Chen X, Li CB, Harrison L, Narashimhan S, Beard CB, Aksoy S: **Tsetse thrombin inhibitor: bloodmeal-induced expression of an anticoagulant in salivary glands and gut tissue of *Glossina morsitans morsitans*.** *Proc Natl Acad Sci U S A* 1998, **95:**14290-14295.

47. Friedrich T, Kroger B, Bialojan S, Lemaire HG, Hoffken HW, Reuschenbach P, Otte M, Dodt J: **A Kazal-type inhibitor with thrombin specificity from *Rhodnius prolixus*.** *J Biol Chem* 1993, **268:**16216-16222.

48. Francischetti IM, Ribeiro JM, Champagne D, Andersen J: **Purification, cloning, expression, and mechanism of action of a novel platelet aggregation inhibitor from the salivary gland of the blood-sucking bug, *Rhodnius prolixus*.** *J Biol Chem* 2000, **275:**12639-12650.

49. Isawa H, Orito Y, Jingushi N, Iwanaga S, Morita A, Chinzei Y, Yuda M: **Identification and characterization of plasma kallikrein-kinin system inhibitors from salivary glands of the blood-sucking insect *Triatoma infestans*.** *FEBS J* 2007, **274:**4271-4286.

50. Morita A, Isawa H, Orito Y, Iwanaga S, Chinzei Y, Yuda M: **Identification and characterization of a collagen-induced platelet aggregation inhibitor, triplatin, from salivary glands of the assassin bug, *Triatoma infestans*.** *FEBS J* 2006, **273:**2955-2962.

51. Noeske-Jungblut C, Kratzschmar J, Haendler B, Alagon A, Possani L, Verhallen P, Donner P, Schleuning WD: **An inhibitor of collagen-induced platelet aggregation from the saliva of *Triatoma pallidipennis*.** *J Biol Chem* 1994, **269:**5050-5053.

52. Glusa E, Bretschneider E, Daum J, Noeske-Jungblut C: **Inhibition of thrombin-mediated cellular effects by triabin, a highly potent anion-binding exosite thrombin inhibitor.** *Thromb Haemost* 1997, **77:**1196-1200.

53. Valenzuela JG, Ribeiro JM: **Purification and cloning of the salivary nitrophorin from the hemipteran *Cimex lectularius*.** *J Exp Biol* 1998, **201:**2659-2664.

54. Ribeiro JM, Schneider M, Guimaraes JA: **Purification and characterization of prolixin S (nitrophorin 2), the salivary anticoagulant of the blood-sucking bug *Rhodnius prolixus*.** *Biochem J* 1995, **308 ( Pt 1):**243-249.

55. Andersen JF, Gudderra NP, Francischetti IM, Valenzuela JG, Ribeiro JM: **Recognition of anionic phospholipid membranes by an antihemostatic protein from a blood-feeding insect.** *Biochemistry* 2004, **43:**6987-6994.

56. Andersen JF, Francischetti IM, Valenzuela JG, Schuck P, Ribeiro JM: **Inhibition of hemostasis by a high affinity biogenic amine-binding protein from the saliva of a blood-feeding insect.** *J Biol Chem* 2003, **278:**4611-4617.

57. Champagne DE, Smartt CT, Ribeiro JM, James AA: **The salivary gland-specific apyrase of the mosquito *Aedes aegypti* is a member of the 5'-nucleotidase family.** *Proc Natl Acad Sci U S A* 1995, **92:**694-698.

58. Faudry E, Lozzi SP, Santana JM, D'Souza-Ault M, Kieffer S, Felix CR, Ricart CA, Sousa MV, Vernet T, Teixeira AR: ***Triatoma infestans* apyrases belong to the 5'-nucleotidase family.** *J Biol Chem* 2004, **279:**19607-19613.

59. Hamasaki R, Kato H, Terayama Y, Iwata H, Valenzuela JG: **Functional characterization of a salivary apyrase from the sand fly, *Phlebotomus duboscqi*, a vector of Leishmania major.** *J Insect Physiol* 2009, **55:**1044-1049.

60. Mans BJ, Coetzee J, Louw AI, Gaspar AR, Neitz AW: **Disaggregation of aggregated platelets by apyrase from the tick, *Ornithodoros savignyi* (Acari: Argasidae).** *Exp Appl Acarol* 2000, **24:**271-282.

61. Stark KR, James AA: **A factor Xa-directed anticoagulant from the salivary glands of the yellow fever mosquito *Aedes aegypti*.** *Exp Parasitol* 1995, **81:**321-331.

62. Perez de Leon AA, Valenzuela JG, Tabachnick WJ: **Anticoagulant activity in salivary glands of the insect vector *Culicoides variipennis sonorensis* by an inhibitor of factor Xa.** *Exp Parasitol* 1998, **88:**121-130.

63. Abebe M, Cupp MS, Ramberg FB, Cupp EW: **Anticoagulant activity in salivary gland extracts of black flies (Diptera: Simuliidae).** *J Med Entomol* 1994, **31:**908-911.

64. Abebe M, Ribeiro JM, Cupp MS, Cupp EW: **Novel anticoagulant from salivary glands of *Simulium vittatum* (Diptera: Simuliidae) inhibits activity of coagulation factor V.** *J Med Entomol* 1996, **33:**173-176.

65. Pereira MH, Souza ME, Vargas AP, Martins MS, Penido CM, Diotaiuti L: **Anticoagulant activity of *Triatoma infestans* and *Panstrongylus megistus* saliva (Hemiptera/Triatominae).** *Acta Trop* 1996, **61:**255-261.

66. Mbow ML, Bleyenberg JA, Hall LR, Titus RG: ***Phlebotomus papatasi* sand fly salivary gland lysate down-regulates a Th1, but up-regulates a Th2, response in mice infected with *Leishmania major*.** *J Immunol* 1998, **161:**5571-5577.

67. Cross ML, Cupp EW, Enriquez FJ: **Differential modulation of murine cellular immune responses by salivary gland extract of *Aedes aegypti*.** *Am J Trop Med Hyg* 1994, **51:**690-696.

68. Ramachandra RN, Wikel SK: **Modulation of host-immune responses by ticks (Acari: Ixodidae): effect of salivary gland extracts on host macrophages and lymphocyte cytokine production.** *J Med Entomol* 1992, **29:**818-826.

69. Gillespie RD, Dolan MC, Piesman J, Titus RG: **Identification of an IL-2 binding protein in the saliva of the Lyme disease vector tick, *Ixodes scapularis*.** *J Immunol* 2001, **166:**4319-4326.

70. Kovar L, Kopecky J, Rihova B: **Salivary gland extract from *Ixodes ricinus* tick polarizes the cytokine profile toward Th2 and suppresses proliferation of T lymphocytes in human PBMC culture.** *J Parasitol* 2001, **87:**1342-1348.

71. Mejri N, Brossard M: **Splenic dendritic cells pulsed with *Ixodes ricinus* tick saliva prime naive CD4+T to induce Th2 cell differentiation in vitro and in vivo.** *Int Immunol* 2007, **19:**535-543.

72. Caljon G, Van Den Abbeele J, Sternberg JM, Coosemans M, De Baetselier P, Magez S: **Tsetse fly saliva biases the immune response to Th2 and induces anti-vector antibodies that are a useful tool for exposure assessment.** *Int J Parasitol* 2006, **36:**1025-1035.

73. Brake DK, Wikel SK, Tidwell JP, Perez de Leon AA: **Rhipicephalus microplus salivary gland molecules induce differential CD86 expression in murine macrophages.** *Parasit Vectors* 2010, **3:**103.

74. Boppana VD, Thangamani S, Alarcon-Chaidez FJ, Adler AJ, Wikel SK: **Blood feeding by the Rocky Mountain spotted fever vector, Dermacentor andersoni, induces interleukin-4 expression by cognate antigen responding CD4+ T cells.** *Parasit Vectors* 2009, **2:**47.
